# Ginsenoside Rg1 delays the senescence of adipose-derived stem cells: network pharmacology and experimental validation

**DOI:** 10.1186/s41065-026-00646-1

**Published:** 2026-01-27

**Authors:** Yuan Fang, Chenghong Mou, Xinlang Yu, Jiakang Zhang, Weina Zhu, Chungen Zhou, Bin Jiang, Yanni Chen

**Affiliations:** 1 Colorectal Surgery Center, Nanjing Hospital of Chinese Medicine Affiliated to Nanjing University of Chinese Medicine, Nanjing, 210022 China; 2 Department of Brain Disease, Nanjing Hospital of Chinese Medicine Affiliated to Nanjing University of Chinese Medicine, Nanjing, 210022 China; 3 Medical Experimental Centre, Central Laboratory, Nanjing Hospital of Chinese Medicine Affiliated to Nanjing University of Chinese Medicine, Nanjing, 210022 Jiangsu China; 4 Department of Biobank, Nanjing Hospital of Chinese Medicine Affiliated to Nanjing University of Chinese Medicine, Nanjing, 210022 Jiangsu China; 5 Jianmin Road Campus, Zhongda Hospital Affiliated to Southeast University (formerly Nanjing Dachang Hospital), Nanjing, 210022 China

**Keywords:** Ginsenoside Rg1, ADSC senescence, Network pharmacology, PI3K/AKT pathway

## Abstract

**Background:**

Mesenchymal stem cell (MSC) senescence limits their therapeutic potential. Adipose-derived stem cells (ADSCs), though easily accessible, are prone to senescence under oxidative or inflammatory stress. Ginsenoside Rg1, with antioxidant and anti-inflammatory properties, may counteract this process. This study investigates whether Rg1 can alleviate ADSC senescence and its underlying mechanisms.

**Methods:**

To explore the molecular mechanisms by which Ginsenoside Rg1 mitigates ADSC senescence, network pharmacology and molecular docking were applied to identify potential signaling pathways and targets. Cell viability was measured using the Cell Counting Kit-8 (CCK-8) assay to select the optimal modeling concentration. Flow cytometry was used to analyze cell cycle distribution and immune-related marker expression in ADSCs. Reverse transcription quantitative PCR (RT-qPCR) quantified stemness- and senescence-related gene expression. Immunofluorescence (IF) staining assessed senescence-associated protein levels. Western blotting (WB) was performed to examine key proteins in the PI3K/AKT signaling pathway.

**Results:**

Network pharmacology identified 22 aging-associated genes targeted by Rg1, with AKT1 emerging as a central hub protein exhibiting high binding affinity. In hydrogen peroxide (H₂O₂)-induced senescence model, Rg1 treatment significantly enhanced cell viability and stemness, while reducing senescence markers p16 and p21. Furthermore, Rg1 attenuated DNA damage, decreased pro-inflammatory cytokine expression, and restored telomere length. It also promoted cell cycle progression and upregulated immune checkpoint proteins CD59 and CD73. Mechanistic analysis revealed that these anti-senescent effects are mediated via activation of the PI3K/AKT signaling pathway.

**Conclusion:**

Rg1 effectively attenuates ADSC senescence by activating the PI3K/AKT signaling pathway, promoting cell cycle progression and enhancing immune evasion capacity. This reveals a novel anti-aging mechanism and offers new insights for MSC-based regenerative therapies, while also providing modern evidence supporting the traditional Jing-Qi theory.

**Supplementary Information:**

The online version contains supplementary material available at 10.1186/s41065-026-00646-1.

## Introduction

MSCs, originating from the mesoderm, are adult multipotent progenitor cells with self-renewal capabilities and the potential to differentiate into various specialized cell types [1]. MSCs were initially isolated from bone marrow aspirates from healthy donors [2], exhibited a stable phenotype, remained as a monolayer in vitro, and could be induced to differentiate into adipocytic, chondrocytic, or osteocytic lineages [3]. Presently, MSCs are isolated from various tissues, including adipose tissue [4], dental tissues [5], skin [6], salivary gland [7], menstrual blood [8], and umbilical cord [9]. ADSCs originate from the stromal vascular fraction (SVF) of adipose tissue [10]. Although constituting only 12–18% of the total adipose cell population, ADSCs distinguish themselves as the most regeneratively potent cell fraction in adipose tissue and are easily accessible [11]. ADSCs are characterized by rapid proliferation and low immunogenicity [10]. Due to the predominantly paracrine nature of these effects, which involve the reduction of inflammation and promotion of tissue repair, ADSCs have been widely utilized in the field of regenerative medicine and cell therapy [12, 13].

MSCs have been extensively utilized in the treatment of a diverse range of diseases [14, 15]. As MSCs age, their regenerative potential diminishes due to changes in cellular functions, including reduced proliferation and differentiation capacities, altered stress-response proteins, and shifts in miRNA expression [16]. However, the journey to successful stem cell therapy requires a veritable multitude of cells, necessitating extensive cultivation in vitro. Given the inherent limitations on MSC proliferation, it is crucial to elucidate the mechanisms driving MSC senescence and develop strategies to counteract these processes [17]. Recent studies have shown that hydrogels as MSC culture carriers [18], along with high-oxygen [19] induction and agents like SS-31 [20], can effectively delay MSC senescence. Among these strategies, traditional Chinese medicine monomers offer promising potential due to their accessibility and efficiency [21].

Huangdi Internal Classic (Huang Di Nei Jing) describes that “Initially, during human development, Jing forms first, and then bones, pulses, tendons, muscles, skin, and hair develop subsequently.” In Chinese Medicine (CM), the major symptoms of aging are considered to be caused by Jing deficiency, especially kidney essence (Shen-Jing) [22]. In CM, MSCs are seen to have self-renewing and multipotent differentiation capacities, which correspond significantly to the properties of Shen-Jing. Panax ginseng C. A. Mey. (Ginseng) effectively replenishes the original Qi. Ginsenoside Rg1, extracted from ginseng, is a prominent natural constituent [23]. According to the theory of Jing-Qi in CM, Rg1 enhances MSC proliferation, effectively retarding their aging process, but the underlying mechanism remains unclear [24].

Accumulating evidence indicates that Rg1 exerts anti-apoptotic and anti-autophagic effects by modulating the PI3K/Akt signaling pathway, which plays a vital role in regulating cellular senescence [25–27]. mTOR serves as a central hub coordinating cell growth, metabolism, and autophagy. The PI3K/Akt pathway functions as a potent upstream activator of mTOR, promoting its activity and driving catabolic processes such as autophagy, thereby accelerating cellular turnover [28]. Therefore, the role of PI3K/Akt dysregulation in aging should be considered within the broader context of its dynamic crosstalk with the mTOR signaling network [29].

Rooted in the network-target theory, network pharmacology elucidates the systemic effects of Traditional Chinese Medicine (TCM) by targeting disease-related biological networks [30]. The use of network pharmacology combined with molecular docking analysis has led to the prediction of ginseng’s anti-aging mechanisms [31]. In this study, we induced senescence in ADSCs using H₂O₂, then treated with Rg1 to assess anti-senescent effects and explore underlying mechanisms.

## Materials and methods

### Network Pharmacology

Aging-related genes were identified by searching “cellular aging” and “cell senescence” in the GeneCards database [32] and the Open Targets Platform [33], and the combined results were used for subsequent analyses.

Kyoto Encyclopedia of Genes and Genomes (KEGG) and gene ontology (GO) enrichment analyses of overlapping gene sets were performed using clusterProfiler v4.10.0, with pathways/terms having adjusted *p* < 0.05 deemed significant [34]. Pathway interrelationships were further explored by constructing enrichment networks in Cytoscape v3.9.0.

### Target prediction of Rg1

Rg1 structure data [35, 36] were obtained from PubChem [37], and targets were predicted using SEA [38], SwissTargetPrediction [39], and SuperPred [40], with predefined filtering criteria. UniProt was used for target annotation [41].

### Protein–Protein Interaction (PPI) network construction and analysis

PPI networks were constructed using STRING v12.0 (interaction score ≥ 0.70; organism: Homo sapiens) [42]. Networks were visualized and topologically analyzed in Cytoscape v3.9.0. The top three hub genes by degree centrality were selected for molecular docking. Corresponding 3D structures were retrieved from PDB or PubChem as appropriate. Docking with Rg1 was performed using AutoDock Vina, and interactions were visualized in PyMOL for validation.

### Culture of ADSCs

ADSCs (cat. no. HUXMD-01001) were purchased from OriCell (China). First-passage cells were maintained in high-glucose DMEM (cat. no. BC-M-005; Biochannel, China) supplemented with 10% FBS (cat. no. BC-SE-FBS01C; Biochannel, China) and 1% penicillin–streptomycin (cat. no. BC-CE-007; Biochannel, China) at 37 °C in a humidified 5% CO₂ incubator. Passage 2 cells were seeded in 6-well plates (2 × 10⁴ cells per well; Costar, Corning, USA) and induced toward osteogenic, adipogenic, and chondrogenic lineages using OriCell differentiation kits (cat. no. HUXMD-90021, HUXMD-90041, HUXMD-90031; China), with medium changes every 2–3 days. After 21 days, cells were stained with Oil Red O, Alcian Blue, and Alizarin Red and observed under a Leica microscope (Germany).

To further confirm MSC identity, ADSCs at passages 3–5 were detached with 0.25% trypsin (cat. no. BC-CE-004; Biochannel; without EDTA and phenol red), centrifuged, and resuspended in PBS containing 0.1% BSA. Cells (1 × 10⁶ per tube) were incubated with antibodies against CD73-APC, CD90-PE, CD105-FITC (positive markers), and CD34-PE, CD45-APC (negative markers) (cat. no. E-AB-F1089E, E-AB-F1226D, E-AB-F1233C, E-AB-F1284D; Elabscience, China; cat. no. 304711, Biolegend, USA) in 100 µL staining buffer for 30 min at 4 °C in the dark. After washing, cells were centrifuged at 1500 rpm for 10 min, resuspended in 500 µL PBS, and analyzed immediately by flow cytometry (Beckman Coulter FC500) with data processed in FlowJo V10. Cells confirmed to meet MSC criteria based on both differentiation potential and surface marker expression were used for all subsequent experiments.

Upon reaching ~ 80% confluence, cells were passaged; passage 5 (P5) cells were used for experiments. The PI3K inhibitor LY294002 (cat. no. HY-10108; MCE, China) was used at a final concentration of 10 µM. The ADSCs were assigned to four experimental groups: (1) Control (CON) group, untreated cells; (2) Model (MOD) group, cells treated with 200 µM H₂O₂ for 24 h to establish the senescence model; (3) Rg1 group, cells treated with 200 µM H₂O₂ for 24 h and co-administered with 30 µM Rg1 to evaluate its protective effect; and (4) LY294002 group, cells pretreated with 10 µM LY294002 for 6 h before co-treatment with Rg1 and H₂O₂ to assess the involvement of the PI3K/AKT pathway.

### Cell viability assay

Cell viability was assessed using the CCK-8 assay (cat. no. C0038; Beyotime, China). P5 ADSCs were seeded in 96-well plates and treated for 24 h with varying concentrations of H₂O₂ (cat. no. 88597; 0–400 µM; Sigma, USA) or Rg1 (cat. no. A10015; 0–50 µM; Shanghai Yuanye, China). After treatment, 10 µL CCK-8 reagent per well was added and incubated at 37 °C for 1–2 h; absorbance at 450 nm was measured for viability assessment. H₂O₂ at 200 µM significantly inhibited ADSC proliferation with relatively low cytotoxicity, while Rg1 at 30 µM significantly promoted cell growth. Therefore, 200 µM H₂O₂ was selected as the optimal modeling concentration, and 30 µM Rg1 was used as the treatment concentration in subsequent experiments.

### Senescence-associated β-galactosidase (SA-β-Gal) staining

SA-β-Gal staining was performed using a commercial kit (cat. no. G1580; Solarbio, China). Cellular senescence was induced in ADSCs by treatment with 200 µM H₂O₂ for 24 h prior to staining. Cells were washed with PBS (cat. no. BC-BPBS-11; Biochannel, China) and fixed in fixative solution for 15 min at room temperature. After a PBS rinse, cells were incubated in SA-β-Gal staining solution at 37 °C overnight without CO₂. Stained cells were imaged on an inverted microscope (Leica, Germany), and positive cells were counted in four random fields per well.

### Quantification of telomere length

Genomic DNA from ADSCs was isolated using the Universal Genomic DNA Purification Mini Spin Kit (cat. no. D0063; Beyotime, China) and adjusted to 5 ng/µL. Cellular senescence was induced by 200 µM H₂O₂ for 24 h prior to DNA extraction. Telomere length was measured by RT-qPCR on an ABI 7500 system using ScienCell’s Absolute Human Telomere Length Assay (cat. no. 8918; USA) [43]. PCR conditions: 95 °C for 10 min, followed by 40 cycles of 95 °C for 20 s, 52 °C for 20 s, and 72 °C for 45 s. Assays were performed in duplicate per kit instructions.

### RT-qPCR

Total RNA was extracted using the FastPure Cell/Tissue Total RNA Isolation Kit V2 (cat. no. RC112-01; Vazyme, China) and reverse-transcribed into cDNA with HiScript III RT SuperMix for RT-qPCR (+ gDNA wiper) (cat. no. R323-01; Vazyme, China). Cellular senescence was induced by 200 µM H₂O₂ for 24 h prior to RNA extraction. Primers (Table 1) were synthesized by GENEray Biotechnology (Shanghai, China). Gene expression was quantified on an Applied Biosystems 7500 Real-Time PCR System (Thermo Fisher Scientific, USA) using ChamQ Universal SYBR RT-qPCR Master Mix (cat. no. Q711-02; Vazyme, China). Relative expression was calculated by the 2^-ΔΔCt method with GAPDH as the reference gene.

**Table 1:** List of RT-qPCR primer sequences

Primer names	Primer sequences
IL-6	F: GAGGTACACGCTCCACACAGAC R: AAGTGCATCGTTGTTCATACA
TNF-α	F: CATCTTCTCAAAATTCGAGTGACAA R: TGGGAGTAGCAAGGTACACCC
IL-1β	F: TGTGAAATGCCACTTTTGA R: GGTCAAAGGTTTGGAGCAG
P16	F: GGGTCGGGTAGAGGAGGTG R: GCTGCCCATCATCATGACCT
P21	F: GTCCTTGGGCTGCCTGTTTT R: GTGGGAAGGTAGAGCTTGGG
OCT4	F: CCTTCGCAAGCCCTCATTTC R: TAGCCAGCTCCGAGGATCAA
Nanog	F: GAATGAAATCTAAGAGGTGGCA R: CCTGGTGGTAGGAAGAGTAAAGG
SOX2	F: AGAACCCCAAGATGCACAAC R: GGGCAGCGTGTACTTATCCT
GAPDH	F: AGAAGGCTGGGGCTCATTTG R: AGGGGCCATCCACAGTCTTC

### WB

Proteins were extracted from ADSCs using RIPA buffer (cat. no. P0013B; Beyotime, China) containing phosphatase and protease inhibitors. The lysates were centrifuged at 12,000 rpm for 15 min at 4 °C, and protein levels in the resulting supernatants were determined using a BCA kit (cat. no. ZJ102; Epizyme Biotech, China). Samples were standardized to 2 µg/µL and denatured at 100 °C for 10 min. Subsequently, 20 µg of protein per lane was separated by SDS-PAGE (cat. no. PG212; Epizyme Biotech, China) and transferred to PVDF membranes (cat. no. IPVH00010; MerckMillipore, USA). After 1 h of blocking, membranes were incubated with primary antibodies targeting TNF-α (1:1000; cat. no. 29652-1-AP; Proteintech, China), p53 (1:1000; cat. no. TA0879; Abmart, China), p-PI3K (1:1000; cat. no. PC6417; Abmart, China), p-AKT (1:1000; cat. no. 4060T; CST, USA), p-mTOR (1:1000; cat. no. 67778-1-Ig; Proteintech, China), total AKT (1:1000; cat. no. 66444-1-Ig; Proteintech, China), PI3K (1:1000; cat. no. 20584-1-AP; Proteintech, China), mTOR (1:1000; cat. no. 66888-1-Ig; Proteintech, China), and GAPDH (1:1000; cat. no. 60004-1-Ig; Proteintech, China), followed by secondary antibody incubation. For investigation of the PI3K/AKT pathway, cells were pretreated with 10 µM LY294002 for 6 h before co-treatment with 30 µM Rg1 and 200 µM H₂O₂. Bands were visualized and analyzed with the ChemiDoc XRS + system (Bio-Rad, USA).

### IF

Cells were washed with PBS, fixed in 4% paraformaldehyde for 30 min at 25 °C, and permeabilized with 0.1% Triton X-100 (cat. no. P0096; Beyotime, China) for 30 min at 4 °C. Cellular senescence was induced by 200 µM H₂O₂ for 24 h prior to IF staining. After blocking with Beyotime P0102 solution for 1 h at room temperature, cells were incubated overnight at 4 °C with primary antibodies against γ-H2AX (1:200; cat. no. 83307-2-RR; Proteintech, China), p16 (1:200; cat. no. 81373-10-RR; Proteintech, China), or p21 (1:200; cat. no. 10355-1-AP; Proteintech, China). Following PBS washes, cells were incubated with Alexa Fluor 488– or FITC-conjugated goat anti-rabbit IgG (cat. no. A0423, A0562; Beyotime, China) for 1 h at 25 °C, counterstained with DAPI for 10 min, and imaged on an Olympus BX43 microscope.

### Cell cycle assay

Cell cycle analysis was performed using the Cell Cycle Staining Kit (cat. no. E-CK-A352, Elabscience, China). Cellular senescence was induced by 200 µM H₂O₂ for 24 h prior to analysis. Cells were fixed in 70% ethanol at 4 °C overnight, washed with PBS, and stained with PI/RNase A solution for 30 min at room temperature in the dark. Cell cycle distribution was analyzed by Beckman Coulter FC500 flow cytometry.

### Flow cytometry

ADSCs at passages 3–5 were detached with 0.25% trypsin, centrifuged, and resuspended in 2 mL ice-cold PBS with 0.1% BSA. Cellular senescence was induced by 200 µM H₂O₂ for 24 h prior to flow cytometry. After two washes at 450 × g for 5 min each, 1 × 10⁶ cells were incubated in 100 µL staining buffer per tube. Then, 5 µL of each antibody (CD73-PE, CD59-APC, cat. no. E-AB-F1242D, Elabscience, China; cat. no. 304711, Biolegend, USA) was added. Samples were incubated for 30 min at 4 °C in the dark. After staining, cells were centrifuged at 1500 rpm for 10 min, washed twice with PBS, and resuspended in 500 µL PBS. Flow cytometry was performed immediately on a Beckman Coulter FC500, and data were analyzed with FlowJo V10.

### Statistical analysis

All experiments were performed independently at least three times. Data are presented as mean ± SD. Statistical comparisons between groups were conducted using one-way ANOVA in SPSS 25.0 and GraphPad Prism 10.0. A *p*-value < 0.05 was considered statistically significant.

## Results

### Rg1 targets aging-related genes via network pharmacology

**Fig. 1:**
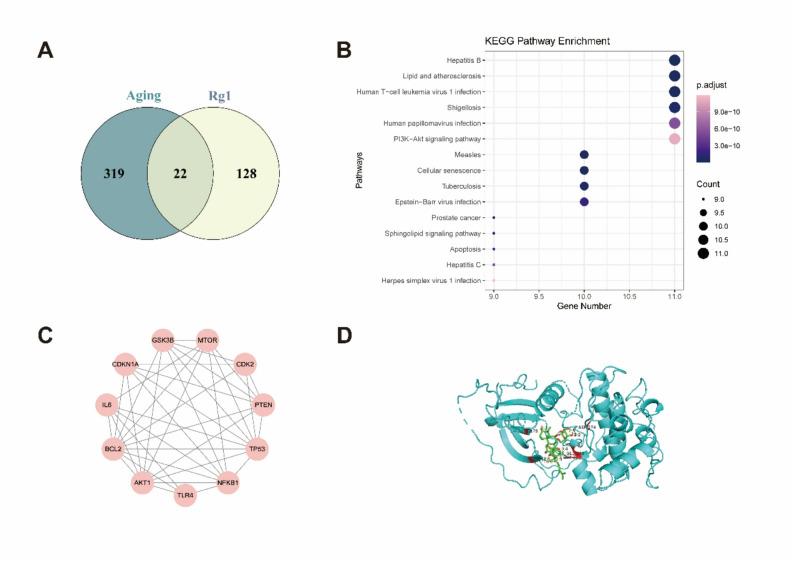
Target prediction via network pharmacology. **A** Venn diagram illustrating the overlap between Rg1-associated targets and ageing-related genes. **B** KEGG pathway enrichment results of the identified hub genes. **C** PPI network of overlapping targets. **D** Binding energy between AKT and Rg1: < − 5 kcal/mol

We identified 150 Rg1-associated targets and 341 aging-related genes from databases, with 22 overlapping genes obtained after standardization using UniProt entries (Fig. 1A). KEGG pathway enrichment revealed that these targets were primarily involved in inflammatory responses, oncogenic signaling pathways, and other disease-associated pathways (Fig. 1B). These genes were analyzed using STRING (score > 0.7) and visualized in Cytoscape to construct a PPI network (Fig. 1C). Molecular docking confirmed a strong interaction between Rg1 and AKT1, with a binding energy less than − 5 kcal/mol (Fig. 1D). Notably, the PI3K/AKT signaling pathway was highlighted as a key axis of regulation.

### Rg1 enhances cell stemness while reducing senescence in H₂O₂-induced senescence model

**Fig. 2:**
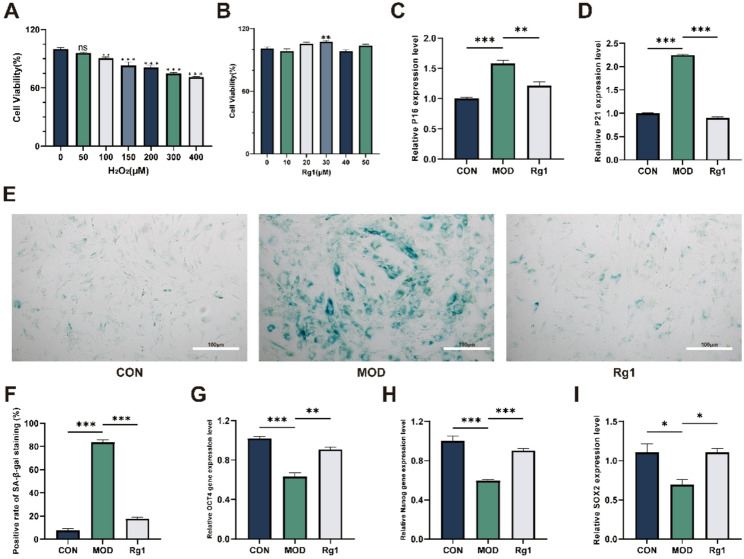
Effects of Rg1 and H₂O₂ on MSC viability, senescence markers, ADSC stemness gene expression, and SA-β-Gal activity in the H₂O₂-induced senescence model. ADSCs were divided into three groups: CON (untreated), MOD (200 µM H₂O₂), and Rg1 (H₂O₂ + 30 µM Rg1). **A**, **B** Cell viability assessed by CCK-8 assay (*n* = 6). Data are presented as mean ± SD. Statistical significance versus untreated ADSCs: *p < 0.05, **p < 0.01, ***p < 0.001; ns, not significant. (**C**, **D**, **G**–**I**) Expression levels of p21, p16, OCT4, Nanog, and SOX2 in the H₂O₂-induced senescence model following co-treatment with Rg1 (*n* = 3). (**E**, **F**) SA-β-Gal assay at 200× (scale bar, 100 μm): cells fixed at RT, stained, and imaged; positive-cell ratio and counts in senescent ADSCs (*n* = 5). Data are presented as mean ± SD; *p < 0.05, **p < 0.01, **p < 0.001 vs. MOD group

All cells used in this study were identified as ADSCs, as confirmed by flow cytometry and trilineage differentiation assays (Fig. S1 A-H). CCK-8 assay showed that H₂O₂ treatment reduced ADSC viability in a dose-dependent manner (Fig. 2A), and 200 µM was chosen for senescence induction. Rg1 at 30 µM significantly enhanced the viability of ADSCs compared with untreated cells (Fig. 2B). Rg1 significantly reduced p21 and p16 mRNA levels (Fig. 2C–D) and SA-β-Gal positivity (Fig. 2E-F) while upregulating OCT4, Nanog, and SOX2 expression (Fig. 2G-I) in the H₂O₂-induced senescence model. These findings support an anti-aging role for Rg1 in ADSCs, via suppression of senescence markers, promotion of stemness genes, and reduction of senescent cell proportion.

### Rg1 alleviates DNA damage and inflammation in H₂O₂-induced senescence model

**Fig. 3:**
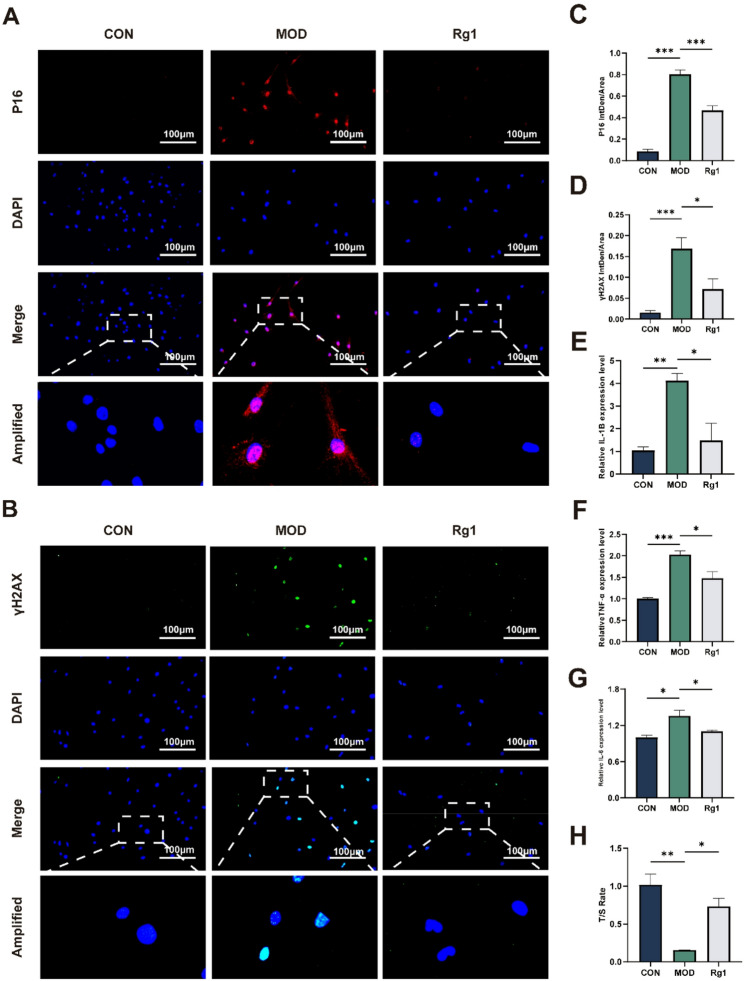
Rg1 reduces both DNA damage and pro-inflammatory cytokine levels in the H₂O₂-induced senescence model. Group abbreviations as in Fig. 2. **A**–**D** IF detection of p16 and γ-H2AX expression in senescent ADSCs (200×; scale bar, 100 μm; *n* = 3). **E**, **F**, **G** qRT-PCR analysis of IL-6, TNF-α, IL-1β mRNA levels (*n* = 3). **H** Relative telomere length (T/S ratio) in each group. Mean ± SD; *p < 0.05, **p < 0.01, **p < 0.001 vs. MOD group

Recognizing DNA damage as a senescence hallmark [44], we used IF to assess p16 and γ-H2AX and RT-qPCR for IL-6, TNF-α and IL-1β mRNA. In addition, relative telomere length was measured to further assess genomic stability. Rg1-treated ADSCs exhibited marked decreases in p16 and γ-H2AX signals (Fig. 3A–D), along with significant reductions in IL-6, TNF-α, and IL-1β expression compared with the model group (Fig. 3E–G). Rg1 significantly elevated the relative telomere length (T/S ratio) in the H₂O₂-induced senescence model (Fig. 3H). These results suggest that Rg1 mitigates H₂O₂-induced DNA damage and suppresses associated inflammatory signaling in ADSCs.

### Rg1 promotes cell cycle progression and restores immune markers

**Fig. 4:**
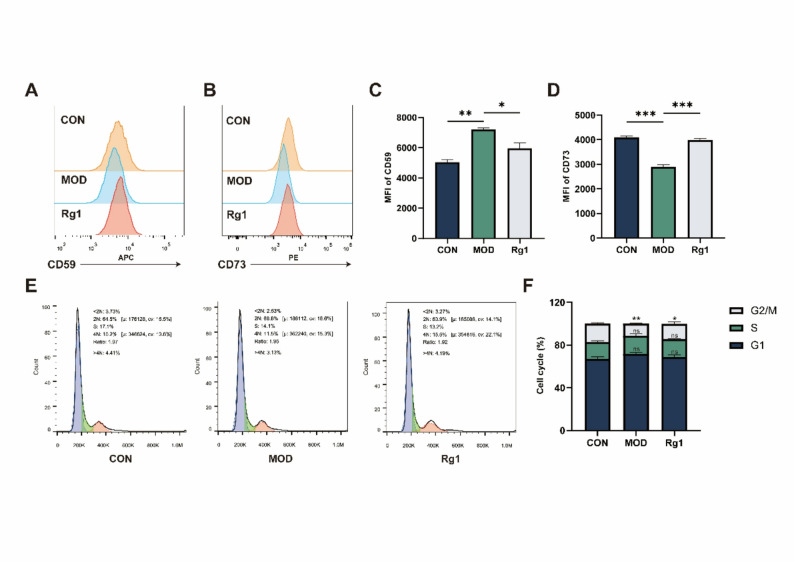
Rg1 impacts cell cycle progression and immune checkpoint proteins in the H₂O₂-induced senescence model. Group abbreviations as in Fig. 2. **A**–**D** Flow cytometry analysis of CD59 and CD73 expression in senescent ADSCs. (*n* = 3). **E**, **F** Cell cycle distribution after Rg1 treatment. (*n* = 3). Mean ± SD; *p < 0.05, **p < 0.01, **p < 0.001 vs. MOD group

CD59 and CD73 serve as critical immune checkpoint molecules that contribute to the immune evasion mechanisms of MSCs [45]. Rg1 effectively restored the expression levels of CD59 and CD73 reduced during H₂O₂-induced senescence (Fig. 4A–D). Cell cycle analysis revealed that, compared with the H₂O₂ group, the proportion of cells in the G0/G1 phase showed a slight decrease without statistical significance, whereas the proportion of cells in the G2/M phase was significantly increased in the Rg1-treated group (Fig. 4E, F). These findings suggest that Rg1 promotes cell cycle progression, modulates MSC immune function, and delays cellular senescence.

### Rg1 delays senescence via PI3K/AKT pathway activation

**Fig. 5:**
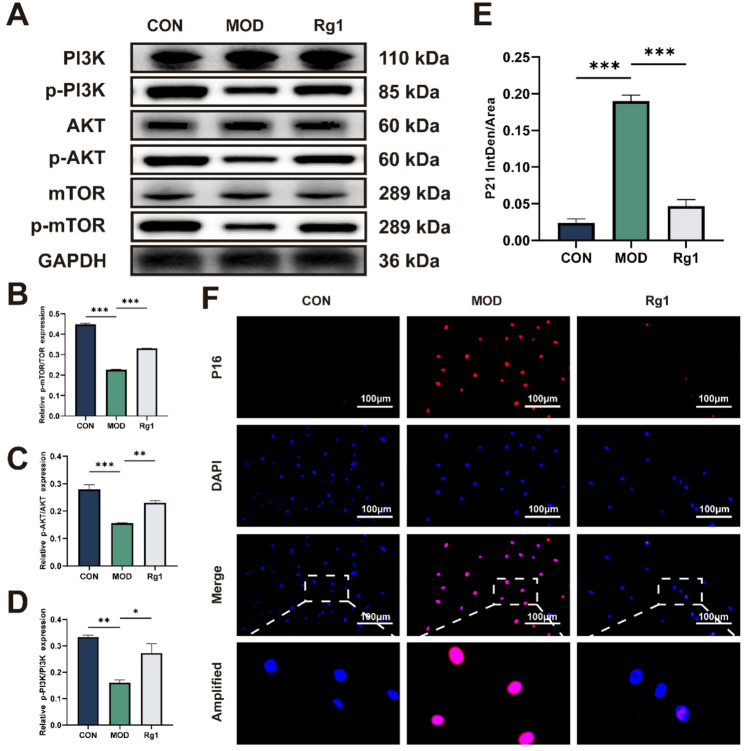
Rg1 delays ADSCs senescence via the PI3K/AKT signaling pathway. Group abbreviations as in Fig. 2. **A**-**D** Representative WB images of p-mTOR, mTOR, p-AKT, AKT, p-PI3K, and PI3K expression in ADSCs (*n* = 4). **E**, **F** IF analysis of p21 levels in H₂O₂-induced senescent ADSCs (200×; scale bar, 100 μm; *n* = 3). Mean ± SD; *p < 0.05, **p < 0.01, **p < 0.001 vs. MOD group

Activation of the PI3K/AKT signaling pathway plays a critical role in preserving cellular function and delaying senescence [46, 47]. H₂O₂ challenge led to led to marked downregulation of p-PI3K, p-AKT, and p-mTOR levels in ADSCs. Rg1 restored these phosphorylation levels, indicating reactivation of the pathway (Fig. 5A–D). Concurrently, nuclear p21 expression in the nucleus was significantly reduced in Rg1-treated ADSCs, as shown by IF (Fig. 5E, F).

### Inhibition of PI3K/AKT abrogates the anti-senescent effects of Rg1

**Fig. 6:**
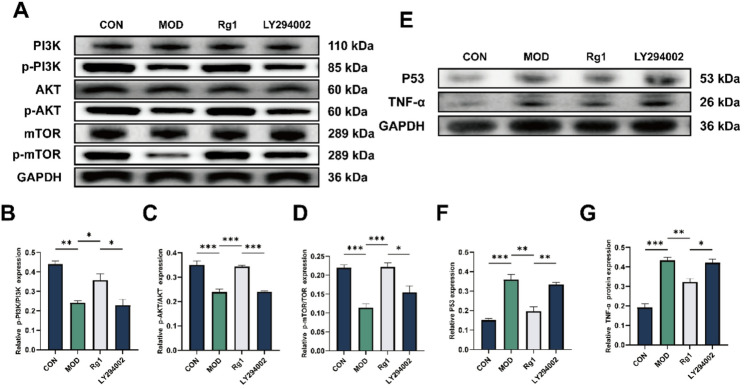
Blocking PI3K/AKT signaling reverses the protective effects of Rg1 against senescence. ADSCs were divided into four groups: CON (untreated), MOD (200 µM H₂O₂), Rg1 (H₂O₂ + 30 µM Rg1), and LY294002 (10 µM LY294002 pretreated for 6 h + H₂O₂ + Rg1). **A**–**G** Western blot detection of PI3K/AKT pathway and senescence/inflammatory markers (p53, TNF-α). Mean ± SD; *p < 0.05, **p < 0.01, **p < 0.001 vs. MOD group

To elucidate whether the PI3K/AKT signaling pathway is essential for the anti-senescent effects of Rg1, we co-treated ADSCs with the PI3K inhibitor LY294002. WB analysis showed that LY294002 decreased the phosphorylation of PI3K, AKT, and mTOR, which was associated with a significant elevation of TNF-α and p53 levels relative to the Rg1-treated group (Fig. 6A–G), suggesting that blockade of PI3K/AKT signaling negates Rg1’s protective effects.

## Discussion

The therapeutic potential of MSC-based transplantation is largely determined by their regenerative and immunomodulatory capacities, which may be compromised by senescence during in vitro expansion or in vivo application [48, 49]. Our study demonstrated that Rg1 effectively delays cellular senescence, consistent with the predictions from network pharmacology. The anti-senescent effects of Rg1 in ADSCs are likely mediated through PI3K/AKT pathway activation, as reflected by reduced SA-β-Gal activity, suppression of p16 and p21, increased pluripotency marker expression, and a lower fraction of G0/G1-arrested cells.

Previous studies have demonstrated that Rg1 alleviates neural stem cell senescence, yet the precise molecular mechanisms responsible for this effect remain unclear [50, 51]. Network pharmacology analysis revealed that Rg1 regulates senescence-associated signaling pathways, particularly PI3K/AKT, while PPI network analysis suggested modulation of key cell cycle regulators, including CCND1, CDKN1A, and CDKN2A. PI3K/AKT signaling plays a crucial role in regulating cellular senescence by promoting the activation of downstream effectors like the p53/p21 axis, thereby maintaining the senescent state [47]. In silico molecular docking further indicated a potential direct interaction between Rg1 and AKT. Supporting this, previous studies have reported that Rg1 activates the PI3K/AKT pathway through the lncRNA-Malat1/miR-124-3p/LAMC1 regulatory axis, thereby promoting astrocyte activation and facilitating spinal cord injury repair [52, 53]. Similarly, cordycepin (COR) has been shown to counteract oxidative stress–induced senescence in bone marrow mesenchymal stem cells through PI3Kα-mediated regulation of PI3K/AKT/FOXO3 signaling [54]. Collectively, these findings support the idea that Rg1 can influence cellular function by modulating the PI3K/AKT pathway, motivating further in vitro studies to assess its capacity to attenuate senescence in adipose-derived mesenchymal stem cells. Our results corroborate the network pharmacology analysis, which proposed that Rg1 exerts anti-senescent effects on MSCs via regulation of PI3K/AKT signaling pathways. To clarify whether PI3K/Akt signaling mediates the therapeutic actions of Rg1, LY294002 [55] was utilized as a pharmacological inhibitor. However, considering its possible non-specific effects, further work is warranted to assess changes in alternative signaling cascades and verify the fidelity of PI3K/AKT suppression.

Senescent stem cells exhibit reduced proliferative capacity, impaired osteogenic differentiation, and weakened immunomodulatory function, characterized by a diminished ability to recruit macrophages and a failure to induce their polarization toward the anti-inflammatory M2 phenotype [56, 57]. Replicative senescence commonly arises during MSC in vitro expansion, resulting in cell cycle arrest, heightened pro-inflammatory signaling, and profound immunomodulatory dysfunction, thereby severely limiting their clinical effectiveness [58, 59]. Increased lysosomal content, a hallmark of senescence [60], reflects the lysosome’s central role in integrating mitogenic and stress cues to regulate metabolism, which can be monitored via SA-β-Gal activity [61]. Rg1 exerts notable anti-senescent effects on MSCs [62]. In H₂O₂-treated ADSCs, it reduced IL-6 and TNF-α secretion, improved cell viability, and suppressed SA-β-Gal expression, reflecting its antioxidant and immunomodulatory properties in line with previous findings. Oct4, Sox2, and Nanog are key transcription factors that sustain pluripotency and self-renewal in stem cells, synergistically regulating multiple target genes [63]. An ethanolic extract from Cucurbita moschata fruit pulp (PKE) has been shown to enhance the stemness of keratinocyte stem cells [64]. Rg1 upregulates Oct4, Sox2, and Nanog expression in H₂O₂-induced senescence model, supporting the role of the Jing-Qi theory in delaying ADSC senescence.

Cellular senescence is a permanent state of cell cycle arrest, characterized by the retention of cells in the G₀ phase and their inability to enter the S phase [65]. Ganoderic acid D (GA-D) has been shown to delay the senescence of bone marrow mesenchymal stem cells (BM-MSCs) in in vivo aging models, with its effects largely attributed to the alleviation of cell cycle arrest, alongside reductions in SA-β-Gal and ROS levels [66]. In H₂O₂-induced senescence model, Rg1 markedly reduced the proportion of mesenchymal stem cells arrested in the G₀ phase, suggesting that Rg1 alleviates senescence-associated cell cycle arrest and enhances the regenerative potential of aged MSCs. In addition, the expression levels of pro-inflammatory cytokines IL-6 and TNF-α were significantly decreased following Rg1 treatment, indicating that oxidative stress–induced inflammatory responses were mitigated. Rg1 has been reported to attenuate ovarian and uterine pathological damage in premature ovarian insufficiency mice via activation of the p21-p53-STK and Bax-Bcl2 signaling pathways [67]. Consistent with these findings, Rg1 treatment in this study led to the downregulation of the cell cycle inhibitors p16 and p21, further supporting its role in alleviating senescence-associated cell cycle arrest.

Accumulation of DNA damage and telomere shortening activate the DNA damage response, leading to irreversible cell cycle arrest and cellular senescence, in which γH2AX serves as a key indicator reflecting DNA damage–associated aging in MSCs [68, 69]. Prior research indicates that mangiferin (MAG) can attenuate MSC senescence and DNA damage triggered by etoposide and high glucose, with a notable reduction in nuclear γ-H2AX levels [70]. Building on these findings, our study demonstrated that Rg1 similarly reduced nuclear γH2AX levels in H₂O₂-induced senescence model, indicating its potential to alleviate DNA damage–associated stem cell aging. Telomeres govern cell fate and aging by sensing replication history and DNA damage, thereby shaping stress and growth responses [71]. Telomere length in MSCs was assessed by RT-qPCR, and results demonstrated significant telomere shortening upon H₂O₂-induced senescence.

**Fig. 7:**
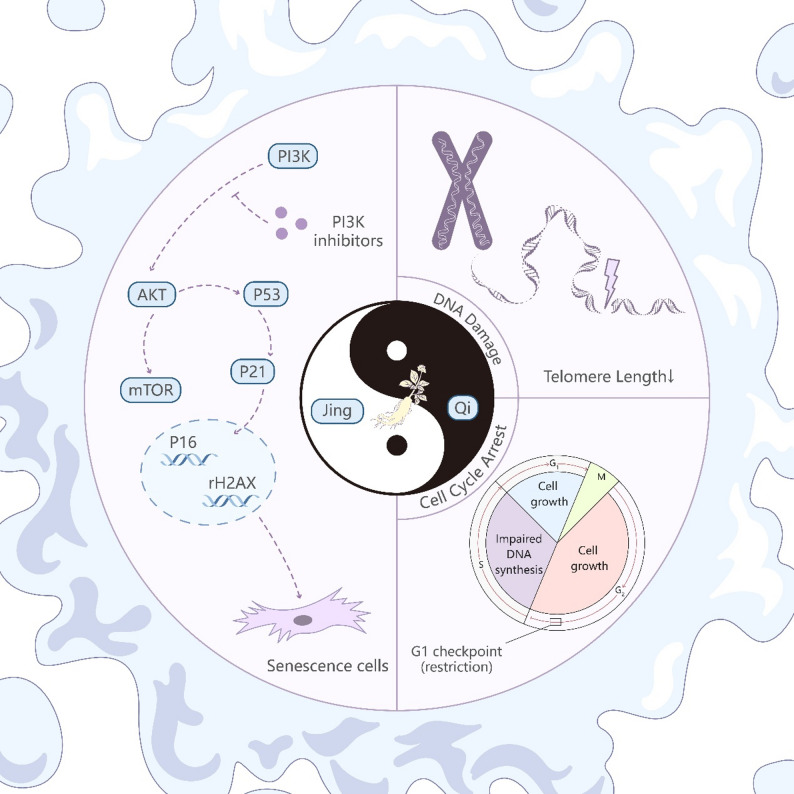
Mechanistic overview of Rg1’s anti-senescence effects in ADSCs via PI3K/AKT activation

TCM can beneficially modulate hematopoietic and endometrial mesenchymal stem cells, enhancing their functional recovery under pathological conditions and supporting therapeutic outcomes [72, 73]. Evidence from both in vitro and in vivo studies highlights TCM’s supportive role in mesenchymal stem cell function (Fig. 7). These results indicate that combining TCM with stem cell therapy, or specifically targeting mesenchymal stem cells with TCM, may provide promising strategies for clinical translation.

## Conclusions

In conclusion, network pharmacology analysis suggests that Rg1 may attenuate ADSC senescence by modulating the PI3K/AKT signaling pathway. In vitro validation demonstrated that Rg1 significantly attenuated senescence in the H₂O₂-induced senescence model, promoted cell cycle progression, maintained low immunogenicity, and reduced DNA damage. These anti-senescent effects are likely mediated through PI3K/AKT-related mechanisms. Collectively, our findings highlight the potential of Rg1 to counteract ADSC senescence and provide mechanistic insights supporting the Jing-Qi theory. Future studies could explore Rg1-based targeted delivery strategies guided by the holistic principles of traditional Chinese medicine to further enhance stem cell transplantation and regenerative outcomes.

## Supplementary Information

Supplementary Material 1.

Supplementary Material 2.

## Data Availability

Supporting data for this study are available from the corresponding author on reasonable request.

## Abbreviations

MSCs: Mesenchymal stem cells
ADSCs: Adipose-derived stem cells
SVF: Stromal vascular fraction
CM: Chinese Medicine
TCM: Traditional Chinese Medicine
H₂O₂: Hydrogen peroxide
PPI: Protein–protein interaction
SA-β-Gal: Senescence-associated β-galactosidase
AMD: Age-related macular degeneration
PKE: Cucurbita moschata fruit pulp
GA-D: Ganoderic acid D
MAG: Mangiferin
GO: Gene ontology
KEGG: Kyoto Encyclopedia of Genes and Genomes
PPI: Protein–Protein Interaction
FBS: Fetal bovine serum
RT-qPCR: Quantitative Real-Time PCR
WB: Western blotting
IF: Immunofluorescence Staining
P5: Passage 5
COR: Cordycepin
