# Supplementary figures and images for: Ginsenoside Rg1 delays the senescence of adipose-derived stem cells: network pharmacology and experimental validation
Source: Hereditas. 2026 Jan 27;163:31. doi: 10.1186/s41065-026-00646-1 (PMC12918114; doi:10.1186/s41065-026-00646-1)

FIG5

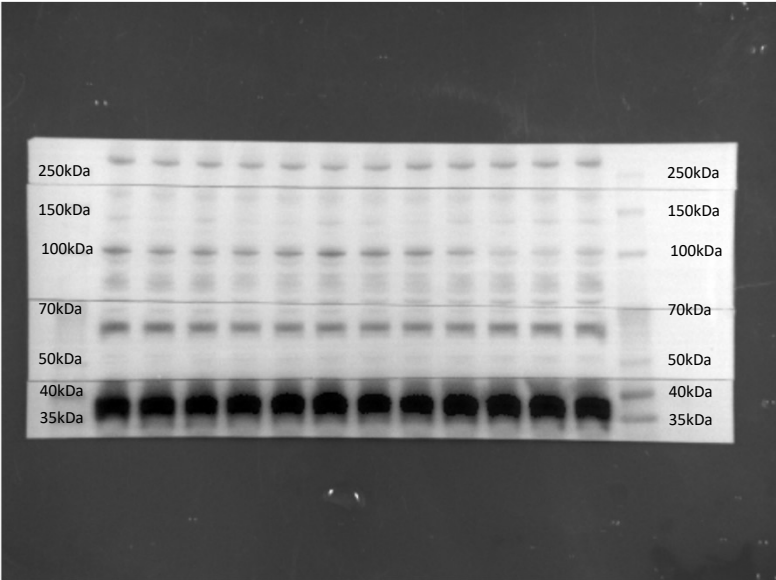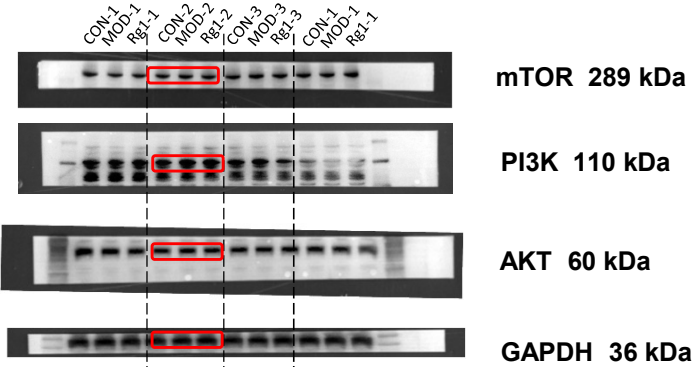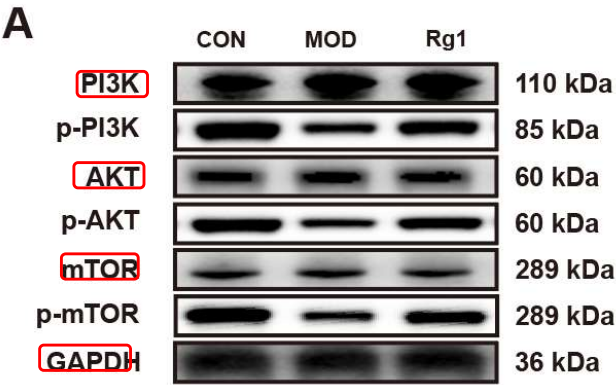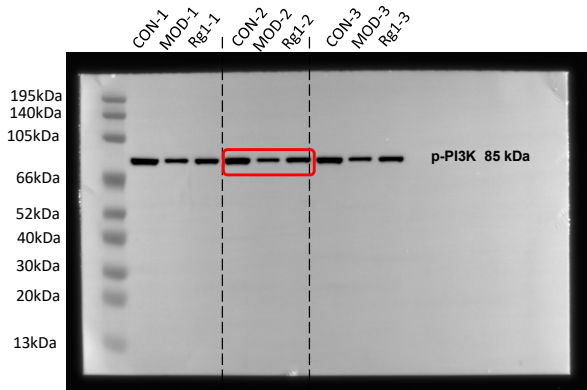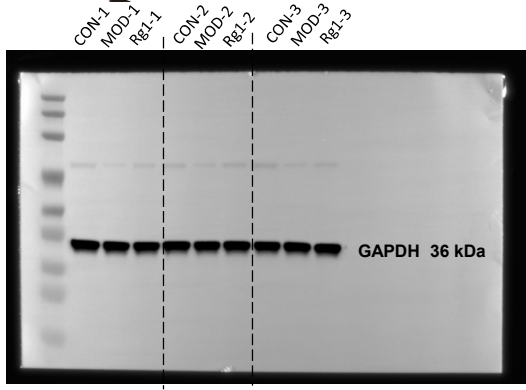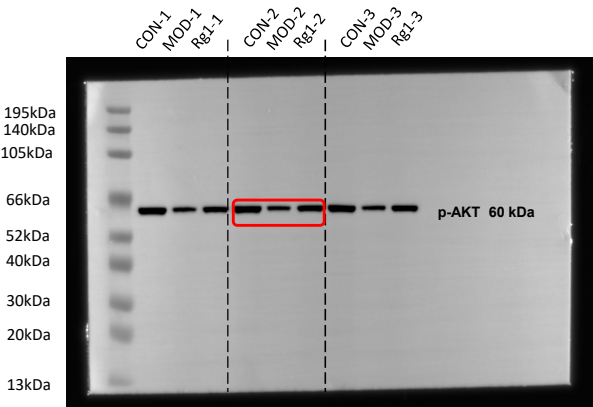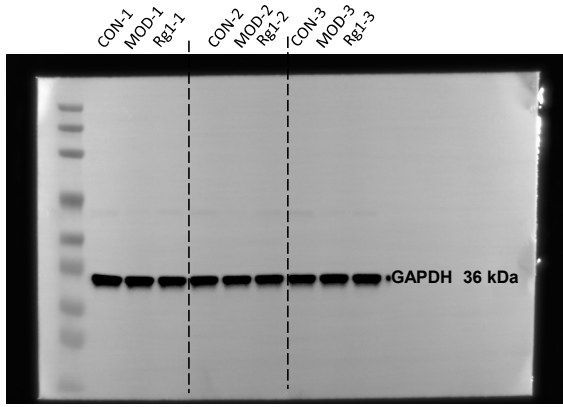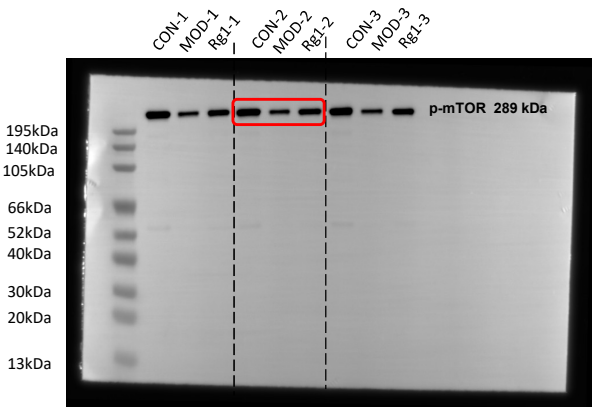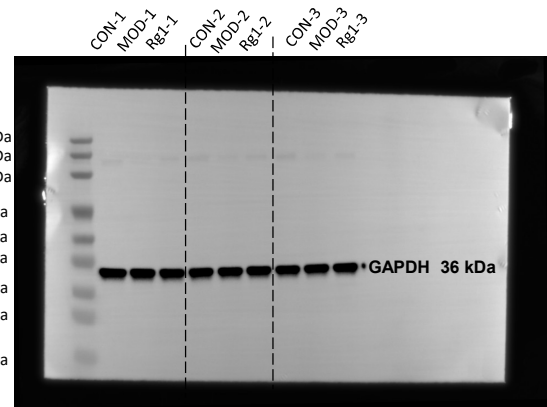

**FIG6**

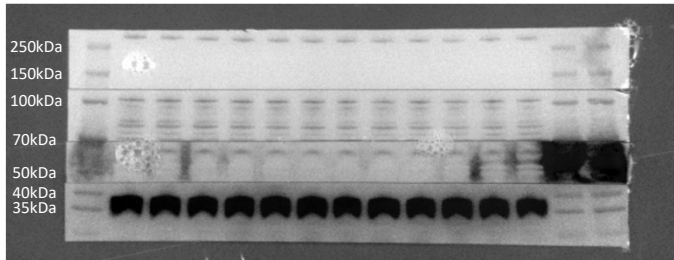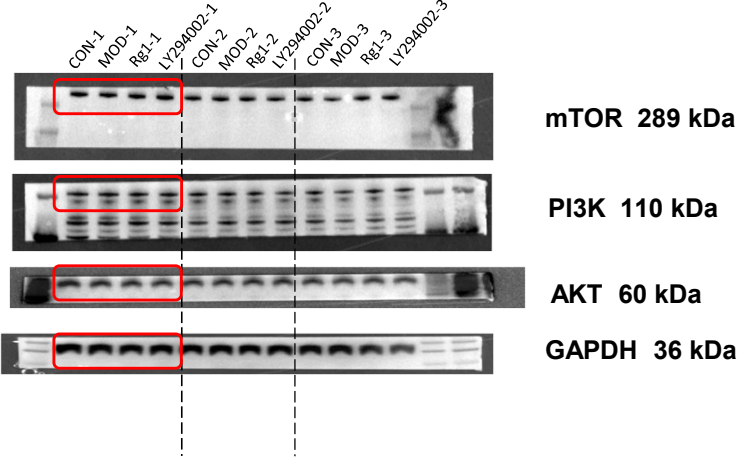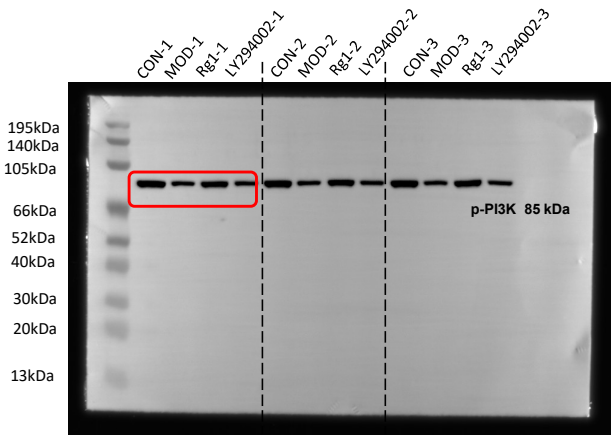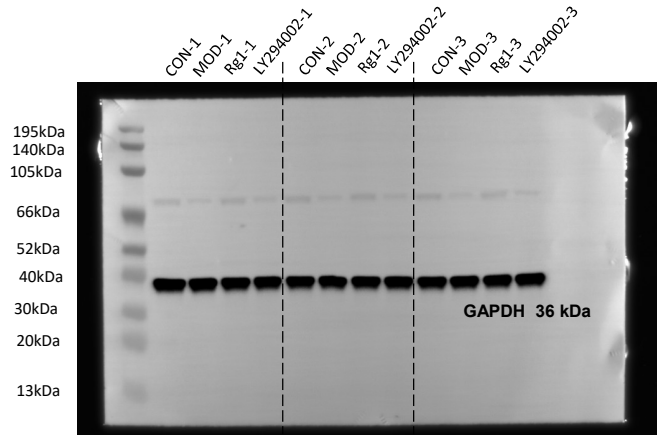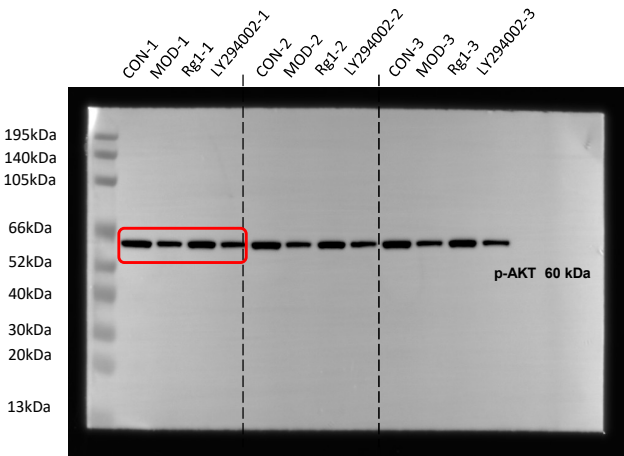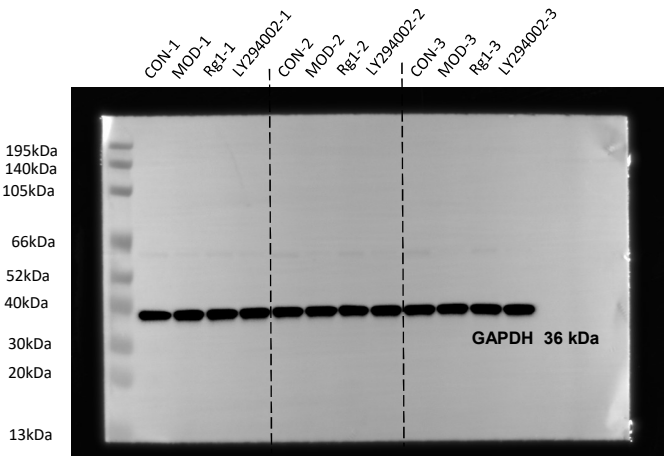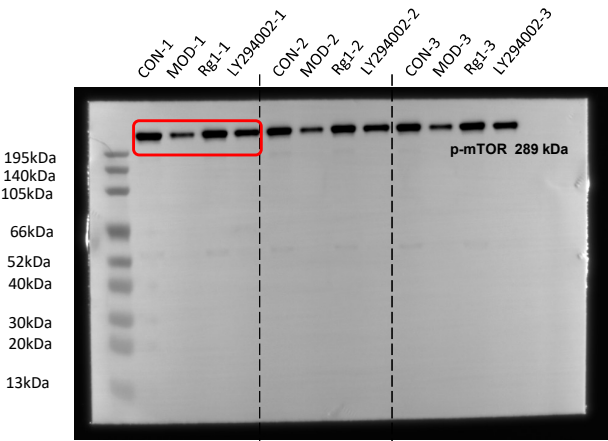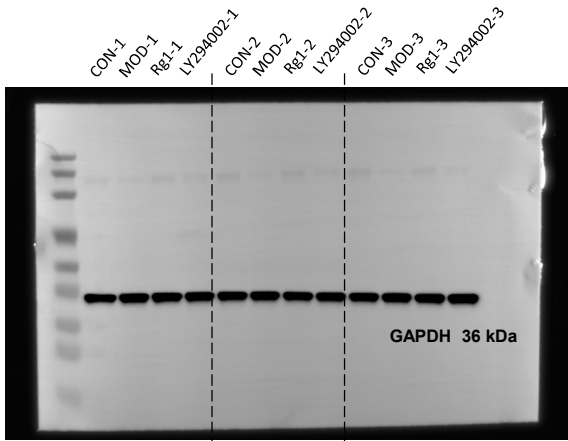

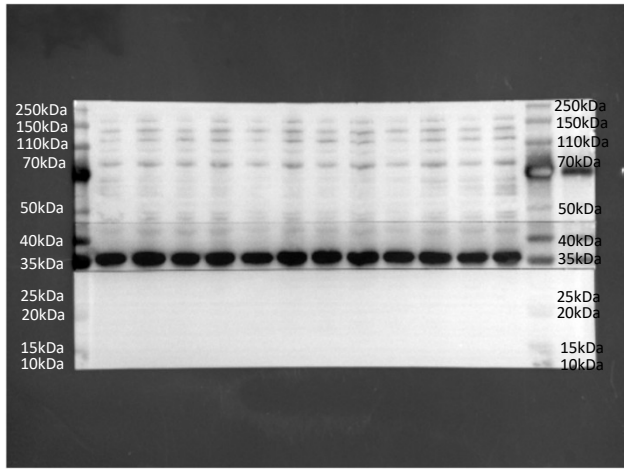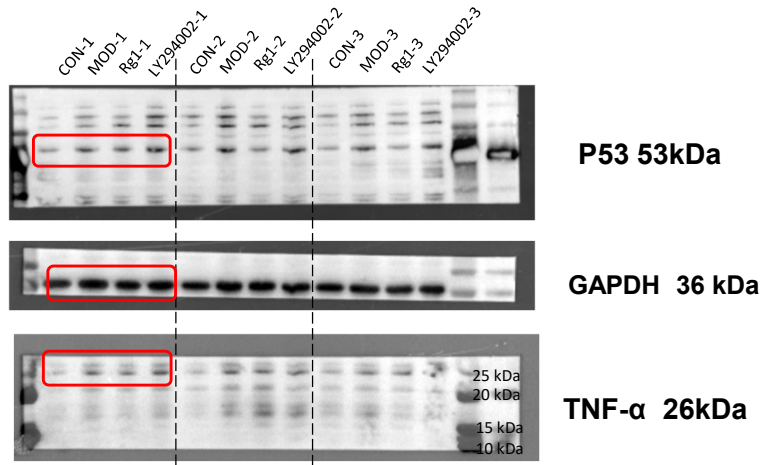

**A**

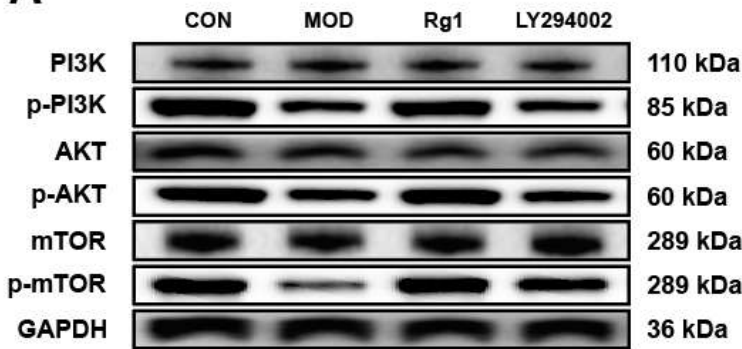

**E**

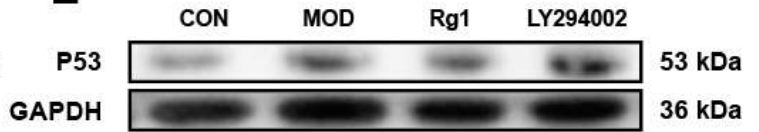

**F**

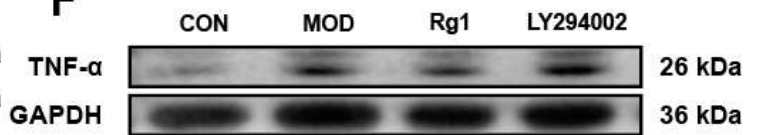

Supplement: Supplementary file 1 — Supplementary Material 1. [file 41065_2026_646_MOESM1_ESM.pdf]
