# Supplementary material for: Ginsenoside Rg1 delays the senescence of adipose-derived stem cells: network pharmacology and experimental validation
Source: Hereditas. 2026 Jan 27;163:31. doi: 10.1186/s41065-026-00646-1 (PMC12918114; doi:10.1186/s41065-026-00646-1)
Supplement: Supplementary file 2 — Supplementary Material 2. [file 41065_2026_646_MOESM2_ESM.docx]

**
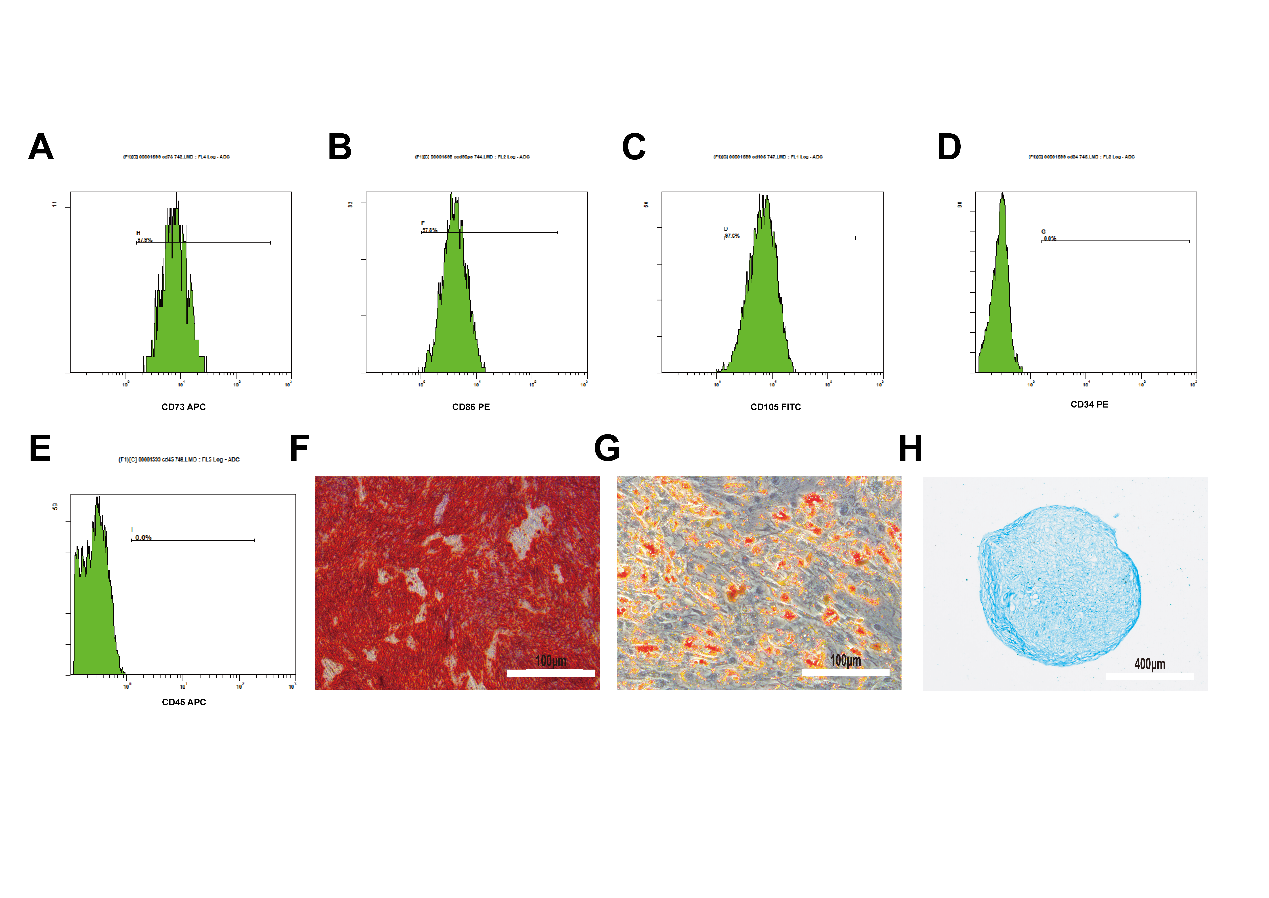
**

**Fig. S1. Validation of MSCs identity.**

(A–E) Flow cytometric assessment of surface antigen profile: MSCs exhibit expected positive expression of CD73, CD90, and CD105, and lack expression of CD34 and CD45. (F–H) Assessment of multilineage differentiation potential after 3-week induction: (F) Oil Red O staining for adipogenic differentiation (200×; scale bar, 100 µm; n = 3); (G) Alizarin Red S staining for osteogenic differentiation (200×; scale bar, 100 µm; n = 3); (H) Alcian Blue staining for chondrogenic differentiation (50×; scale bar, 400 µm; n = 3).
